# A multi-channel stimulator with an active electrode array implant for vagal-cardiac neuromodulation studies

**DOI:** 10.1186/s42234-024-00148-3

**Published:** 2024-07-06

**Authors:** Fangqi Liu, Maryam Habibollahi, Yu Wu, Nazanin Neshatvar, Jiaxing Zhang, Ciro Zinno, Outman Akouissi, Fabio Bernini, Lisa Alibrandi, Khatia Gabisonia, Vincenzo Lionetti, Jacopo Carpaneto, Henry Lancashire, Dai Jiang, Silvestro Micera, Andreas Demosthenous

**Affiliations:** 1 Department of Electronic and Electrical Engineering, University College London, Torrington Place, London, WC1E 7JE UK; 2 BioRobotics Institute, Scuola Superiore Sant’Anna (SSSA), 56025 Pisa, Italy; 3 EPFL, Campus Biotech, CH-1202 Geneva, Switzerland; 4 BioMedLab, Scuola Superiore Sant’Anna (SSSA), Pisa, Italy; 5 Department of Medical Physics and Bioengineering, University College London, Gower Street, London, WC1E 6BT UK

**Keywords:** Active electrode array, Implant fabrication, Integrated stimulator, Microelectrodes, Neuroprosthesis, Vagus nerve stimulator

## Abstract

**Background:**

Implantable vagus nerve stimulation is a promising approach for restoring autonomic cardiovascular functions after heart transplantation. For successful treatment a system should have multiple electrodes to deliver precise stimulation and complex neuromodulation patterns.

**Methods:**

This paper presents an implantable multi-channel stimulation system for vagal-cardiac neuromodulation studies in swine species. The system comprises an active electrode array implant percutaneously connected to an external wearable controller. The active electrode array implant has an integrated stimulator ASIC mounted on a ceramic substrate connected to an intraneural electrode array via micro-rivet bonding. The implant is silicone encapsulated for biocompatibility and implanted lifetime. The stimulation parameters are remotely transmitted via a Bluetooth telemetry link.

**Results:**

The size of the encapsulated active electrode array implant is 8 mm × 10 mm × 3 mm. The stimulator ASIC has 10-bit current amplitude resolution and 16 independent output channels, each capable of delivering up to 550 µA stimulus current and a maximum voltage of 20 V. The active electrode array implant was subjected to in vitro accelerated lifetime testing at 70 °C for 7 days with no degradation in performance. After over 2 h continuous stimulation, the surface temperature change of the implant was less than 0.5 °C. In addition, in vivo testing on the sciatic nerve of a male Göttingen minipig demonstrated that the implant could effectively elicit an EMG response that grew progressively stronger on increasing the amplitude of the stimulation.

**Conclusions:**

The multi-channel stimulator is suitable for long term implantation. It shows potential as a useful tool in vagal-cardiac neuromodulation studies in animal models for restoring autonomic cardiovascular functions after heart transplantation.

## Background

Orthotopic heart transplantation is considered the last resort for end-stage heart disease. Since the surgery involves the complete removal of the native heart, denervation is inevitable regardless of the applied surgical techniques. Consequently, the life quality of patients is limited due to unpredictable changes in the modulation of the heart rate and the load-contractility relationship of the donor heart. The vagus nerve (VN) modulates vital functions including respiration, blood circulation and digestion (Johnson and Wilson 2018). VN stimulation of cervical segments has shown potential for the treatment of chronic heart failure (Klein and Ferrari 2010). The development of a cardiac neuroprosthesis based on VN stimulation is a promising approach to restoring the functionality of the denervated heart (Strauss et al. 2023).

Physiological studies (Aukrust et al. 2005; Wang et al. 2004; Champion et al. 2004) suggested that the parasympathetic system is involved in the regulation of endothelial nitric oxide expression, and dysregulation of nitric oxide pathways. It was found that a reduced vagal ganglionic transmission impairs contractility and cardiac function and leads to progression of heart failure (Sabbah et al. 2011). Also, parasympathetic activity can inhibit inflammatory cytokine release and may help to prevent tissue injury and cell death by its anti-inflammatory response. However, as there are many afferent and efferent nerves which pass through this VN segment, care must be taken not to stimulate off target nerve fibers in error as this may adversely affect immunity (Yuan and Silberstein 2015) or central neuroplasticity (Meyers et al. 2018).

For successful heart denervation treatment an implantable multi-channel VN stimulator is required, where for high selectivity the electrodes need to be placed accurately at the target area (Beekwilder and Beems 2010; Meyers et al. 2018; Vallone et al. 2021; Cracchiolo et al. 2021). As treatment progresses, the stimulation patterns (e.g. the pulse shape, and frequency in individual channels) (Vallone et al. 2021) should be adjusted accordingly to adapt to the evolving condition of the patient. Therefore, for each channel, the stimulator should provide multiple adjustable waveform parameters to provide customized stimulation and study the effect of adjusting the stimulation patterns. Moreover, the cardiac-vagal neuroprosthesis should be small and robust for long term implantation.

Implantable systems for VN or peripheral nerve stimulation have been reported (Li et al. 2022; Habibagahi et al. 2022; Shah et al. 2022). In (Li et al. 2022) a closed-loop VN stimulator for refractory epilepsy treatment is presented. It is wirelessly powered and provides EEG (electroencephalogram) recording and 4-channel VN stimulation. The VN stimulator in (Habibagahi et al. 2022) has two stimulation channels for multisite modulation. Another miniaturized blood pressure neuromodulation system is presented in (Shah et al. 2022). This low power consumption peripheral nerve stimulation implant delivers controlled stimulus current between 90 µA to 750 µA and shows promising results in acute testing.

**Fig. 1:**
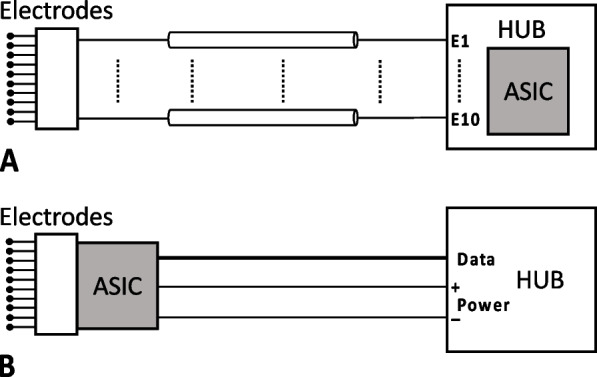
Two methods of connecting stimulation electronics to implanted electrodes. **A** Stimulator ASIC placed in a hub at a distance away from the electrodes. This passive electrode approach requires many cables between the hub and the implanted electrodes. **B** Stimulator ASIC placed very close to the electrodes. This active electrode approach greatly reduces the cable count to the hub

Although reported systems are capable of VN stimulation, the main challenge when applying precise VN stimulation for long term cardiovascular treatment is not fully addressed. To achieve targeted stimulation for cardiac functions with minimum side effects to other organs, it is preferable to place the electrodes at the thoracic level of the VN. On the other hand, it is optimal to implant the stimulator electronics close to the skin for more reliable transcutaneous power transfer and communication. This results in long electrode leads between the stimulation sites and the electronics. Conventional pacemakers adopt this arrangement, but they have a limited number of stimulation channels and therefore few conducting cores in the lead. In multi-channel VN stimulators, however, it is preferable to have a high electrode count for precise neural intervention and minimal side effects. As the number of electrodes increases, the electrode leads become cumbersome (see Fig. 1A).

A multi-channel VN stimulator with active electrodes (where the electronics are located close to the electrodes, see Fig. 1B) would help to solve the issues and reduce inter-channel crosstalk (Jiang and Demosthenous 2018). As shown in Fig. 1B, since many electrode channels can now be digitally controlled, fewer cables for power and data transmission are required between the active electrodes and the hub, thus the electrode count can scale up without increasing the number of cables. Systems with a large number of stimulation channels have been reported (Liu et al. 2012; Giagka et al. 2015a, b; Liu et al. 2022). The distributed stimulation system in (Liu et al. 2012) was designed to drive multiple implantable electrodes. However, the control of the multiple channels is slow as each electrode must be addressed in sequence, requiring many instructions to be sent to each individual electrode. An active electrode array for epidural spinal cord stimulation was presented in (Giagka et al. 2015a, b) with successful acute testing. However, the stimulator is placed outside the active electrode and the stimulation channels cannot be controlled independently.

This paper presents a 16-channel stimulation system with an active electrode array implant for vagal-cardiac neuromodulation studies in swine species. The implant has a custom-designed, low-power stimulator application specific integrated circuit (ASIC) (Wu et al. 2021) that is mounted on a ceramic substrate and connected to an intraneural electrode array (Strauss et al. 2023) via rivet bonding. For precise stimulation each channel can be addressed via a serial peripheral interface (SPI) providing independently controlled stimulations. The active electrode array implant is percutaneously connected to an external hub, which provides the power supply and data settings to the stimulator ASIC. The stimulation parameters are remotely transmitted via a Bluetooth low energy (BLE) telemetry link. By reducing the number of cables, the active electrode minimizes the system failure rate. To verify the feasibility of using the active electrode array implant as a neural stimulation interface, preliminary in vivo testing was performed on the sciatic nerve of a Göttingen minipig. The electromyogram (EMG) response elicited was recorded and quantified to evaluate the implant performance when changing stimulation parameters. The rest of the paper describes the design, fabrication, and testing (electrical, in vitro and in vivo) of the implantable VN stimulation system.

## Methods

### Design constraints

The volume of an implant for in vivo animal studies must consider the size of the animal and the target nerve, in order to minimize both adverse effects on the animal’s quality of life and damage to the nerve and the surrounding tissue during surgery. In this application, the target VN fibers are deeply rooted near the pig’s heart. The larger the incision, the greater the risk of damage and infection to surrounding tissue, nerves, and the arteries. The dimension of the implant should be minimized to reduce the incision size.

Power consumption is another important implant design consideration. Low power consumption reduces heat dissipation, reducing the risk of damage to tissue and nerves surrounding an implant. The acceptable temperature increase of an implant is within 2 °C (BS EN 45502–1:2015, 2015). For an implant with active electrodes, the power consumption of the stimulator ASIC should be minimized as it will be implanted closer to the target nerve.

Device lifetime is important for long term implantation. Generally, physiological studies need prolonged experiments to examine both the effectiveness and any side effects of neural stimulation. The overall testing procedure often takes weeks. To effectively study the functionality of vagal-cardiac neuromodulation in heart transplantation, interfascicular regenerative electrodes are used, requiring a period of several weeks for adequate nerve regeneration to ensure a good connection between the electrodes and nerve. An implant for use with such electrodes must be appropriately encapsulated to prevent electrical failures.

**Fig. 2:**
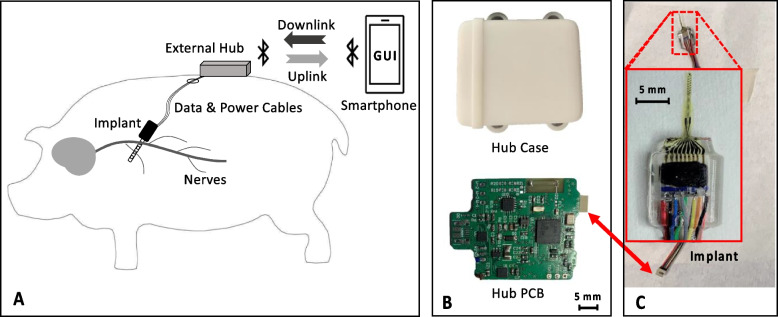
Implantable stimulation system for vagal-cardiac neuromodulation studies in swine species. **A** Showing the overall structure of the stimulation system, comprising an active electrode array implant, an external wearable hub and a smartphone with a custom GUI. The smartphone communicates with the external hub via Bluetooth. The external hub and the implant are connected percutaneously with cables that provide power to the implant and a bidirectional data link. **B** The 3D printed case of the external hub (upper figure) for mounting on the back of the pig to protect the hub PCB (lower figure). **C** The active electrode array implant and the cables to the external hub

The overall structure of the implantable VN stimulation system is shown in Fig. 2A. The active electrode array implant is percutaneously connected to an external wearable hub, which provides the power supply and sends stimulation parameters to the stimulator ASIC. Although developing a fully implantable system for VN stimulation would help to reduce the risk of infection in long term use (Liu et al. 2022), integrating low power delivery and versatile stimulation control requires further circuit development. To minimize the risk of failure during long term use in the animal, a percutaneous connection was used between the external hub and the active electrode array implant. Since the target nerve fibers are located near the pig’s heart, 100 cm long cables were used to facilitate placement of the active electrode array implant near the target nerve, as shown in Fig. 2C. The hub was designed to be mounted on the pig’s back (as it is hard to be accessed by the animal); for protection it was enclosed in a 3D printed case (see Fig. 2B). The stimulation parameters are remotely controlled by a smartphone via the BLE link over a distance between 2–3 m.

### Stimulator ASIC

**Fig. 3:**
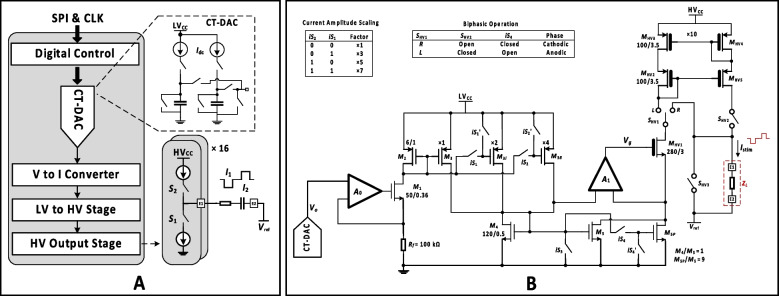
Details of the stimulator ASIC. **A** The architecture of the stimulator ASIC. **B** Transistor level details of the output stage of the stimulator ASIC for delivering biphasic stimulus currents (*I*_stim_) to the electrodes

To address the power constraint discussed earlier, a low power stimulator ASIC (Wu et al. 2021) was used. Figure 3A shows the architecture of the ASIC. It was designed in a 0.18 µm CMOS high voltage (HV) technology and features a novel capacitor-charge time-based digital-to-analog converter (CT-DAC). The circuit comprises 16 independent stimulation channels, each capable of delivering up to 550 µA stimulus current with a HV output stage that can be operated up to 20 V. The stimulator is based on a source-sink current driver topology and has low crosstalk which is important for multi-channel stimulation.

A key feature of the stimulator ASIC is the CT-DAC offering about 10-bit current amplitude resolution with low power consumption. As shown in Fig. 3A, by controlling the charging period of a capacitor, the desired output voltage level, previously supplied by a conventional current-DAC with multiple current branches, can instead be generated by simply charging the capacitor with a small constant dc current (*I*_dc_). Hence, the CT-DAC significantly reduces the quiescent current. After voltage-to-current (V to I) conversion, the signal is coupled to a HV output stage for stimulation, leading to a linear time-to-current conversion scheme which can be easily controlled by the digital counter.

The stimulator ASIC has 16 identical output stages. Figure 3B shows the transistor level details of the output stage. The CT-DAC is placed on the low voltage (LV) side and the source of transistor *M*_1_ is clamped at higher voltage levels, which leads to a wider dynamic range for the CT-DAC. The drain current of *M*_1_ can be scaled with different factors by controlling the switches of the intermediate stages (*M*_2_, *M*_3_, *M*_3i_, *M*_3ii_). For a cathodic phase (current from electrode E2 to E1) of a biphasic pulse, the HV switch *S*_HV1_ is connected to position *R*, providing the cathodic current via transistors *M*_5_ and *M*_5P_. For the anodic phase (current from E1 to E2), switch *S*_HV2_ is closed, *S*_HV1_ is connected to position *L*, and current is provided via the HV PMOS current mirror (*M*_HV2_ to *M*_HV5_). The gate of *M*_5P_ is grounded (by switching on *iS*_4_), and the drain current of *M*_HV1_ is equal to that of *M*_4_. It is scaled up by a factor of 10 by the *M*_HV2_—*M*_HV5_ current mirror to save power in the *M*_HV1_ branch. To discharge the electrodes, *S*_HV3_, *iS*_3_ and *iS*_4_ are closed.

### Active electrode array implant design

**Fig. 4:**
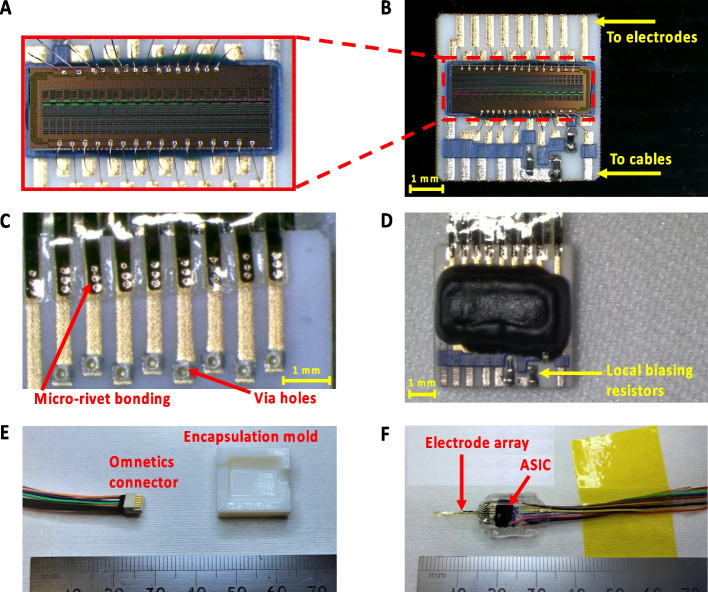
Fabrication of the active electrode array implant. **A** Detailed view of the wire-bonded stimulator die. **B** Integration of the stimulator die on the ceramic substrate to create the active implant. **C** Detail view of the rivet bonding process. **D** Overview of the active implant before silicone encapsulation. **E** Omnetics connector with cables and the 3D printed half mold for encapsulation. **F** Oblique view of the encapsulated active electrode array implant

The stimulator die (see Fig. 4A) was mounted on a ceramic substrate that acts as an adaptor between the cables and the intraneural electrode array (Strauss et al. 2023). As shown in Fig. 4B, a two-sided thick film substrate was screen printed (Custom Interconnect Ltd, UK) onto a 550 µm alumina ceramic; 250 µm diameter vias were laser drilled and filled with PdAg; Au tracks were printed to all wire bond sites; PtAu was overprinted at all solder sites; dielectric was overprinted at the ASIC die attach location and at any interface between Au tracks and PtAu solder sites. The ASIC was adhered to the substrate and gold wire bonded. Wire bonds were protected with glob top epoxy (Eccobond 50,400–1). Local biasing resistors were soldered to the ceramic substrate close to the ASIC die to minimize temperature differences. Interconnects were formed between the electrode array and the ceramic substrate by gold micro-rivet bonding (Lancashire et al. 2019; Stieglitz et al. 2000) on both sides of the substrate. In order to successfully attach the bonding ball to the Au coating surface, it is critical that the bonding surface is clean. Before micro-rivet bonding, the tracks were cleaned using acetone isopropyl alcohol, and deionized water to remove soldering flux and other contaminations. As shown in Fig. 4C, for each stimulation channel three bonding balls were placed to ensure both good electrical and mechanical connections. The cables were soldered to the PtAu solder sites using Hydro-X solder. The fabricated active implant has dimensions of 7.1 mm × 7.2 mm × 2 mm (Fig. 4D).

### External hub design

**Fig. 5:**
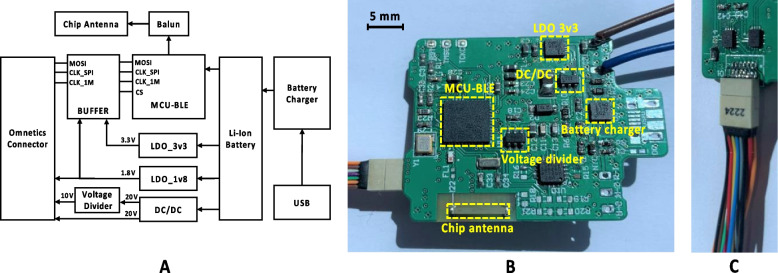
External hub design. **A** System architecture of the external hub. **B** Detailed view of the external hub PCB with various components highlighted. **C** Detailed view of the Omnetics cable connector

Figure 5A shows the system architecture of the external hub and Fig. 5B shows the hub’s fabricated printed circuit board with key components highlighted. The hub provides power and stimulation control to the active electrode array implant. As discussed earlier, placing the external hub on the pig’s back minimizes the risk of device damage caused by animal movement. The hub is powered by a rechargeable Li-ion battery of 4300 mAh capacity, sufficient for at least one week of device operation. A battery recharging module (LTC4079, Analog Devices Inc., USA) recharges the battery at 500 mAh; (a tradeoff between charging time and heat generation). A 3.3 V low-dropout (LDO) regulator powers a microcontroller unit (MCU), and a 1.8 V LDO regulator provides the LV supply to the stimulator ASIC. The impedance of the electrodes is in the range of a few kΩ. A DC-DC converter (DC/DC) with a 20 V output ensures sufficient stimulation compliance voltage when delivering stimulus currents above 200 µA. Since the stimulator is based on a current source and sink topology, the compliance voltage is further divided by a voltage divider to provide a 10 V reference voltage.

For data transmission, a BLE-based wireless MCU (CC2640 Texas Instruments, USA) manages the wireless data telemetry between the smartphone and the external hub. The uplink transmits to the smartphone the hub’s battery level and the status of the BLE connection, whilst the downlink transmits the stimulation parameters to the hub. The stimulation parameters are sent to the active electrode array implant from the MCU via a 16 bit, 8 MHz SPI. An Omnetics connector (Fig. 5C) with cables (A78620-001 Omnetics, USA) connects the active electrode array implant and hub. Using 100 cm length cables allows to position the implant close to the target nerve during the surgical operation. However, long cables increase the load impedance and the I/O pins of the MCU have limited driving capability. In order to minimize the effect of load impedance variations on the SPI data transmission, a digital buffer with dual-bit bus (SN74AVC4T245, Texas Instruments, USA) was used. The buffer is supplied by both 1.8 V and 3.3 V, which helps to down convert the 3.3 V SPI signal of the MCU to 1.8 V for the LV supply of the stimulator ASIC. The buffer has a maximum data transmission rate of 380 Mbps, sufficient for the 8 MHz SPI.

### Communication protocol and GUI

**Fig. 6:**
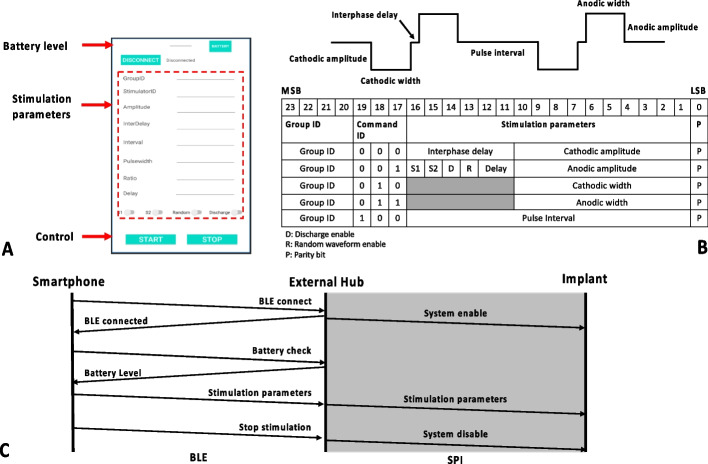
Details of the GUI and communication protocol. **A** Smartphone based GUI for controlling the implant and stimulation parameters. **B** Specifications of the biphasic stimulus current pulse (upper) and details of the communication protocol (lower table) for programming the stimulus pulses. **C** Details of the communication process between the smartphone and the active electrode array implant

Communication between the smartphone and the active electrode array implant is relayed by the external wearable hub. The wireless link to the external hub also manages the BLE connection and monitors the hub’s battery level. An Android-based graphical user interface (GUI) was developed using Kotlin (SKD, version 29) and installed on the smartphone to allow programming of the stimulation parameters. After pairing the smartphone with the hub via BLE, the active electrode array implant can be controlled from the GUI. The GUI design is shown in Fig. 6A. The progress bar on the top monitor indicates the battery level of the hub. The type-in text boxes (marked with red dot line) are used to program the stimulation waveform (as shown in Fig. 6B top panel). On pressing the start button, the stimulation parameters are wirelessly transmitted to the hub using eleven 8-bit data frames. The MCU reframes the received data into the format shown in Fig. 6B. Each data frame comprises 24 bits. The highest 3 bits are allocated to the group ID, used to group multiple channels together for synchronized stimulation. Stimulation from different groups is independent from each other. The subsequent 3 bits are the command ID for distinguishing the commands. The rest of the bits define the details of the stimulation waveform as shown in Fig. 6B. The least significant bit is a parity bit for error detection. The GUI packages the setting parameters into eight 8-bit data frames. They are transmitted to the MCU in the hub via BLE. The MCU repackages the data into a 16-bit vector and transmits it to the active electrode array implant via 16-bit SPI. The complete communication process between the smartphone and the active electrode array implant is shown in Fig. 6C.

### Implant encapsulation

Before encapsulation, the active electrode array implant was carefully cleaned to ensure good adhesion with the encapsulating silicone, which was applied for implant biocompatibility and robustness for long term implantation (Vanhoestenberghe and Donaldson 2013). The implant substrate was cleaned by sequential ultrasonic washing in deionized water, acetone, and deionized water. The substrate was then encapsulated with a two-part, medical grade, low viscosity, optically clear, silicone elastomer (MED-6015, AvantorNusil, Radnor, PA, USA). MED-6015 Part A and Part B were mixed in a ratio of 10:1 in a speed mixer for 3 min at 2500 rpm (Dual Asymmetric Centrifugal Laboratory Mixer System, DAC 150 FVZ-K, Synergy Devices Ltd, UK). A half mold was designed in Autodesk CAD (2022) and was 3D printed using verowhite (opaque polyjet resin) as shown in Fig. 4E. The size of the mold in Fig. 4E is 18 mm × 12 mm × 3 mm, allowing the active implant to be surrounded by silicone rubber of thickness 1–1.5 mm. Before encapsulation, the mold was cleaned in deionized water and acetone with ultrasonication. During encapsulation the substrate was placed with the stimulator ASIC facing the bottom of the mold and was supported by pre-formed silicone spacers to ensure the ASIC was fully encapsulated. The silicone rubber was allowed to cure at room temperature (21 °C) for at least 48 h. The total size of the encapsulated part of the active electrode array implant shown in Fig. 4F is 8 mm × 10 mm × 3 mm.

### Accelerated lifetime testing

**Fig. 7:**
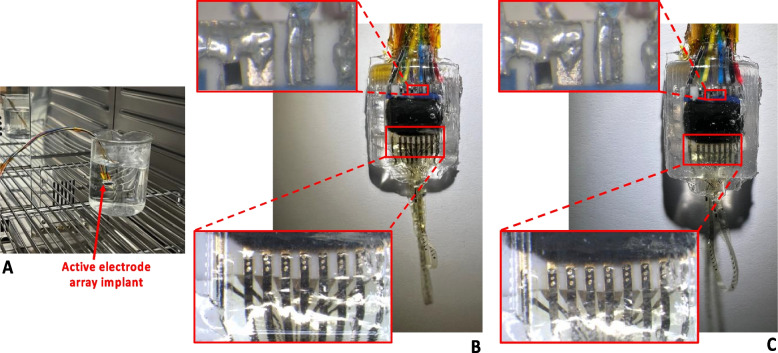
Accelerated lifetime test of the encapsulated active electrode array implant. **A** The implant immersed in deionized water in a climate chamber at a constant temperature of 70 °C for 7 consecutive days. **B** Zoomed-in view of the active electrode array implant prior to the test. **C** Zoomed-in view of the active electrode array implant after 7 days in the chamber showing no corrosion on the tracks, soldering joints and micro-rivet bonding sites

The reliability of the active electrode array implant for long-term use was evaluated with accelerated lifetime testing. The test setup is shown in Fig. 7A. The implant was placed in a beaker filled with deionized water. The consideration of using deionized water is that silicone rubber has low permeability to metal salts (Donaldson et al. 2011) and high permeability to water vapor, so the most likely failure mode is due to the penetration of moisture through the encapsulation layer (Donaldson 1996). Should there be any ionic contamination on the surface of the implant (underneath the silicone encapsulation layer), then the osmotic gradient drives water molecules toward the contaminant and contributes to the formation of a pocket of liquid water.

The beaker was placed in a climate chamber (HPP110eco, Memmert GmbH, Germany) at a constant temperature of 70 °C for 7 consecutive days. The humidity of the climate chamber was set to 80% to minimize the volume change of the deionized water. The implant was constantly powered and was frequently monitored from the hub during the 7-day test period. Figure 7B shows the zoomed-in view of the active electrode array implant prior to the test. At the end of the 7-day test, no corrosion was observed on the tracks, soldering joints and micro-rivet bonding sites of the electrodes, as shown in Fig. 7C. The active electrode array implant was tested in vitro in saline solution (0.9%) both prior to, and after, the 7-day test. The results are reported in “Stimulation outputs in saline” below. There was no change in the electrical function after the accelerated lifetime test.

### Safety of implant surface temperature

**Fig. 8:**
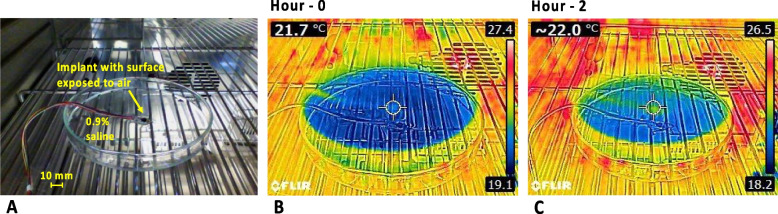
Evaluation of the surface temperature of the encapsulated active electrode array implant. **A** The implant immersed in 0.9% saline solution inside a climate chamber, with the upper surface exposed to air for accessing changes to its surface temperature; **B** Thermal image of the implant prior to testing shows a surface temperature of 21.7 °C; **C** Thermal image of the implant after 2 h continuous stimulation shows 0.3 °C change in the surface temperature

The surface temperature of the implant due to heat dissipation by its electronics was evaluated. As shown in Fig. 8A the implant was placed in a petri dish filled with 0.9% saline solution, whose thermal conductivity (0.6 W/m/°C) is close to that of blood (0.52 W/m/°C).^1^ The upper surface of the implant was exposed to air to allow direct access to an infrared thermal camera. The surface temperature test was conducted inside the climate chamber so that the ambient temperature remained constant. The chamber temperature was set to 23 °C. Before the measurement, the setup was placed in the chamber for 1 h to allow it to stabilize to the ambient temperature. The surface temperature of the implant was measured using a FLIR E4 thermal imaging camera (FLIR systems, Wilsonville, OR, USA) with the camera perpendicularly focused on the exposed area of the implant. Changes in the surface temperature of the implant were measured after 2-h continuous delivery of biphasic stimulus current pulses (200 µA amplitude, 50 µs width, 10 ms pulse interval). As shown in Fig. 8B and C, the surface temperature of the implant after 2-h operation increased 0.3 °C, from the level prior to the test. The test was repeated three times to minimize environmental variation and instrument measurement error. The three temperature changes observed were 0 °C, 0.3 °C, and 0.4 °C. The measured temperature changes are all within the 2 °C safety range stated in EN 45502–1 (BS EN 45502–1:2015, 2015) for long term implantation.

### Stimulation outputs in saline

**Fig. 9:**
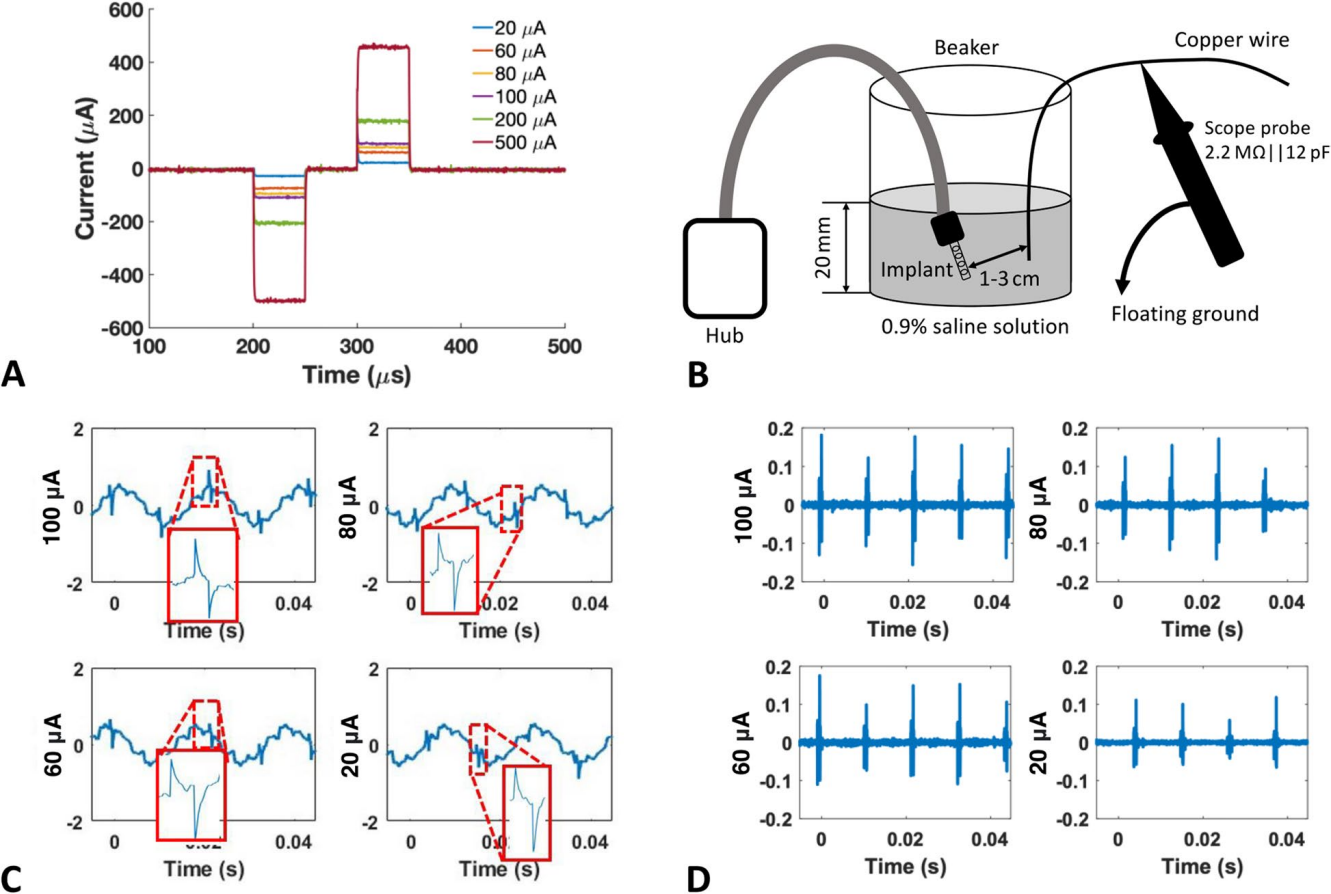
Evaluation of the stimulation outputs. **A** Stimulation outputs measured with a 12.4 kΩ dummy load resistor. **B** In vitro test setup for measuring the stimulation outputs of the encapsulated active electrode array implant in 0.9% saline solution. **C** In vitro stimulation outputs with 100 μA (scope scale 500mV/ 5ms), 80 μA (scale 500 mV/ 5 ms), 60 μA (scale 500 mV/ 5 ms), and 20 μA (scale 500 mV/ 5 ms) pulses, 50 µs pulse width in each phase, and 10 ms pulse interval. **D** In vitro stimulation waveforms after high pass filtering in Matlab (first-order filter with a cut-off frequency of 40 Hz)

To verify the stimulator circuits, the output from the stimulator ASIC on the active electrode was measured before bonding the intraneural electrode array onto the substrate. A 12.4 kΩ resistor was used as a dummy load to represent the impedance of the electrode as reported in (Strauss et al. 2023). The stimulation pulse amplitude was set to 500 µA, 100 µA, 80 µA, 60 µA and 20 µA. The measured current pulse waveforms are shown in Fig. 9A.

After encapsulation, the active electrode array implant was placed inside a beaker filled with 0.9% saline solution at 23 °C. The external hub was placed outside the beaker with the cables connected to the implant. The test setup is shown in Fig. 9B. Only approximate stimulation measurements could be recorded because the implant was fully encapsulated and the electrodes were sub-mm size, too small for the available probing methods. The electrode array delivered controlled current pulses to the saline solution. A copper ‘pick-up’ wire was placed inside the beaker. Voltage changes in the saline due to the current pulses from the stimulator were measured from the pick-up wire using an oscilloscope probe with the ground of the probe floating. The reason for using a single pick-up wire instead of two wires for differential recording is that the stimulating and return electrodes were separated within only a few hundred micrometers on the electrode array.

Figure 9C and D show the measured stimulation outputs with the current level set to 100 µA, 80 µA, 60 µA and 20 µA, with 50 µs pulse width in each phase, 50 µs interphase delay, and 10 ms interval between pulses. The measured waveforms in Fig. 9C show clear 50 Hz interference from the power mains due to the floating probe arrangement. However, the biphasic pulses of the simulation output can still be seen, with the zoomed-in view of the stimulation pulses shown in red squares. The raw recorded waveform was processed in Matlab (Mathworks, Natick, MA, USA) with a second-order high pass filter (40 Hz cut-off frequency) to reduce the interference noise and the results are shown in Fig. 9D. It is evident that the measured stimulation amplitude increases with higher current level.

This test was repeated on the implant after it had undergone accelerated lifetime testing. The measured results were identical to those in Fig. 9C, indicating that the implant remained functional after the accelerated lifetime test.

### In vivo results

#### Surgical preparation

**Fig. 10:**
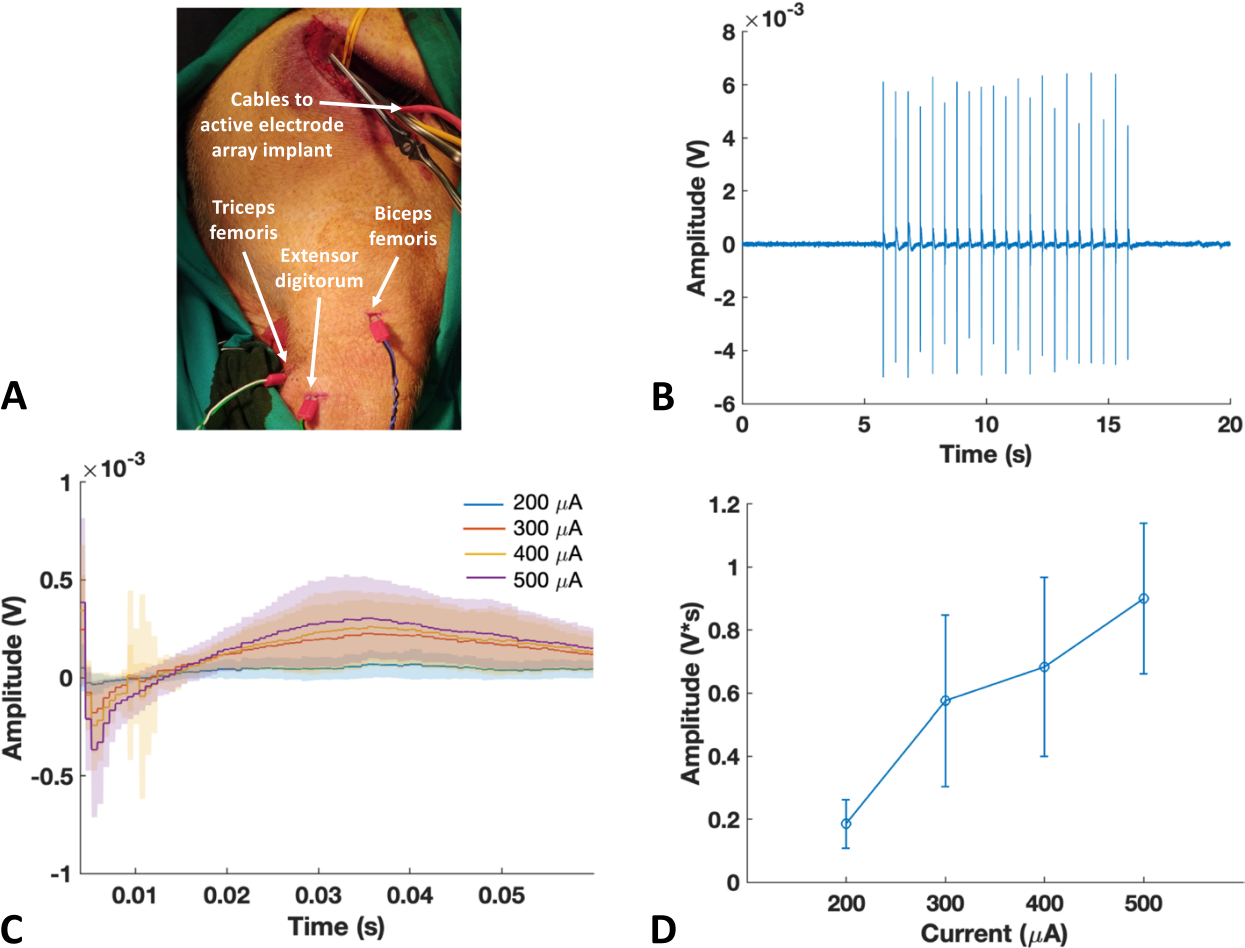
EMG response upon single-channel sciatic nerve stimulation. **A** Experimental setup: the intraneural electrode array was implanted in the sciatic nerve, and the stimulator was lodged in a pocket under the animal’s skin. The three needle EMG electrodes were implanted on the thigh, with a differential configuration. **B** Recording session from the fingers’ extensor muscle upon sciatic nerve stimulation, with visible stimulation artifact and EMG response afterwards. **C** Magnification on the extensor digitorum EMG response with varying stimulation amplitudes. **D** Area under the absolute value of the EMG response increasing with varying stimulation amplitudes

The protocol for all animal studies (no. 76/2014 PR) was approved by the Italian Ministry of Health and was in accordance with Italian law (D.lgs. 26/2014). One healthy adult male Göttingen minipig (Ellegaard Göttingen Minipigs A/S, Dalmose, Denmark; body weight 35 kg) was used in this study. The animal was pre-medicated using Zoletl® (10 mg/kg) and Stressnil (1 mg/kg). The minipig underwent anesthesia with propofol (2 mg/kg intravenously) followed by maintenance with 1–2% sevoflurane in oxygen-enriched air (50%) during mechanical ventilation (Ferraro et al. 2021; Lionetti et al. 2013). Throughout the experiments, the animal received an infusion of 500 mL NaCl (0.9%) solution to manage intraoperative fluid. A fluid-filled catheter was inserted in the carotid artery to measure arterial blood pressure, and ECG (electrocardiogram) was continuously monitored. An approximately 8-cm long cut was performed on the skin between the biceps femoris and gluteus superficialis muscles. The connective tissue was removed to gain access to the sciatic nerve through a retractor. Two rubber strings (Mediloops, Neuromedex, Germany) were used to isolate the nerve without causing any damage. The intraneural electrode array was implanted in the sciatic nerve transversally, and the stimulator was lodged in a pocket created under the skin. Three needle EMG electrodes were implanted percutaneously by making a 4 mm cut on the skin to facilitate the insertion. The electrodes were implanted in three different muscles: biceps femoris, triceps femoris, and extensor digitorum (Fig. 10A).

#### Data collection and analysis

Recordings of 20 s were taken, each consisting of 5 s baseline EMG activity, 10 s intraneural stimulation, and 5 s of washout. Differential recordings of the EMG signal were obtained from the needle EMG electrodes. All the recordings were performed using a TDT system (Tucker Davis Technologies; Alachua, FL, USA) at 24k samples per second. Data processing and analysis were carried out in Matlab. EMG data was filtered using a bandpass filter between 10 Hz and 800 Hz. The timing of each stimulation pulse was obtained by identifying the stimulation artifacts. The integral of the absolute value of the EMG response was calculated in a 130 ms window after the stimulation artifact, to include the fastest and the slowest response.

#### Stimulation parameters

Cathodic-first, charge balanced, square waves were delivered with intensities between 200 µA and 500 µA, frequency 2 Hz, and pulse width 200 µs. The stimulation was delivered using single channels of the intraneural electrode array (active sites 1–16). The amplitude of the stimulation was increased from 200 µA to 500 µA in three steps (100 µA each), in different recording sessions.

#### EMG response upon sciatic nerve stimulation

Figure 10B shows the EMG response of the extensor digitorum EMG signal upon sciatic nerve stimulation with the maximal amplitude (500 µA) using a single channel. To further validate the stimulator coupled with the intraneural electrode array, an amplitude sweep (200:100:500 µA) was performed. The resulting EMG response is shown in Fig. 10C, with increasing stimulation amplitude. Similarly, Fig. 10D shows that the area of the absolute value of the EMG response increases with increasing injected current.

## Discussion

Table 1 provides a comparison of state-of-the-art active neural interface stimulation systems. The table demonstrates the tradeoff between system functionality (hence complexity) and power consumption. Compared to other active neural interfaces, the active electrode array implant in this work supports the largest number of stimulation channels with the highest resolution of stimulus in a wide range of stimulation intensities, with minimal power consumption. Although free-floating implantable systems, such as the ones in (ElAnsary et al. 2021) and (Piech et al. 2020), have a very small surgical footprint, the anatomical location of the target nerve, which is deep inside the rib cage, imposes significant challenge to wireless power delivery. The active electrode array implant in this work offers localized independent multi-channel stimulation compared with (Giagka et al. 2015a, b; Lancashire et al. 2019) and (Piech et al. 2020), and consumes significantly less power than (Liu et al. 2012; ElAnsary et al. 2021) and (Ballini et al. 2017), hence less heat dissipation to the nerve.

**Table 1:** Comparison with other implantable stimulation systems

	**This work**	(Shah et al. 2022)	(Habibagahi et al. 2022)	(Liu et al. 2022)	(Jia et al. 2020)	(Lancashire et al. 2019)	(Giagka et al. 2015a, b)
Nervous system target	Vagus nerve	Vagus nerve	Vagus nerve	Peripheral nerve	Peripheral nerve	Peripheral nerve	Spinal nerve
Electrode type	Active	Passive	Passive	Passive	Passive	Active	Active
Stimulation channels	16	1	2	4 × 4 × 4	8	7 ✕ 7	13
Implant dimensions	8 × 10 × 3 mm^3^	4.5 × 18.5 × 13.4 mm^3^	20.1 mm^3^	40 × 20 × 10 mm^3^	70 × 50 × 30 mm^3^	^a^2.9 × 1.38 mm^2^	^a^8.67 mm^2^
SoC power consumption	23.3 μW	N/A	27 μW	28 μW	20.7 mA	149 μW	114 μW
Maximum current	500 μA	750 μA	3.3 V/1.8 V (constant voltage based)	16 mA	770 μA	500 μA	1 mA
Output current resolution	10-bit	8-bit	Constant voltage	11-bit	5-bit	Externally supplied	Externally supplied

^a^Dimensions of a single ASIC

Swine sciatic nerve stimulation in acute settings was performed to validate the performance of the device. For in vivo test, Fig. 10 demonstrates that a single channel effectively elicited an EMG response that grew progressively stronger with increasing stimulation amplitude. The EMG response shows a first sharper peak representing the quick muscle contraction, followed by a slower response. The thin-film polyimide intraneural electrode array was also previously characterized in vitro and in vivo in the swine VN by the authors (Agnesi et al. 2023; Strauss et al. 2023). Together, these results suggest the possibility of modulating VN activity using this device.

## Conclusions

An implantable 16-channel neuroprosthesis for vagal-cardiac stimulation has been developed as a research tool into chronic heart denervation treatment in swine species. To minimize the risk of failure in chronic implantation, the multi-channel stimulator ASIC and microelectrode array are integrated into an active electrode array implant, thereby reducing the number of implantable interconnections from the external hub to the intraneural electrode array. The implant stimulation parameters are remotely controlled via a BLE data telemetry link with low power consumption. The implant has been encapsulated in silicone for biocompatibility and electronics protection. The methods of fabrication of the implant have been described. The functionality of the implant has been examined through electrical, surface temperature and accelerated lifetime testing in vitro. The results demonstrate the device feasibility for long term implantation. In addition, the preliminary in vivo test conducted verified the feasibility of using the active electrode array implant as a neural stimulation interface. Future work will entail VN in vivo testing and further implant development.

## Data Availability

The experimental data is available from the corresponding author on reasonable request.

## Abbreviations

ASIC: Application specific integrated circuit
BLE: Bluetooth low energy
CMOS: Complimentary metal-oxide semiconductor
CT-DAC: Capacitor-charge time-based digital-to-analog converter
ECG: Electrocardiogram
EEG: Electroencephalogram
EMG: Electromyogram
GUI: Graphical user interface
HV: High voltage
ID: Identity document
LV: Low voltage
LDO: Low dropout
MCU: Microcontroller unit
PMOS: P-channel metal-oxide semiconductor
SPI: Serial peripheral interface
VN: Vagus nerve

## Footnotes

1 IT'IS Foundation, Tissue Properties. https://itis.swiss/virtual-population/tissue-properties/database/thermal-conductivity/

## References

[CR1] Agnesi F (2023). Cardiovascular response to intraneural right vagus nerve stimulation in adult minipig. Neuromodulation.

[CR2] Aukrust P (2005). Inflammatory and anti-inflammatory cytokines in chronic heart failure: Potential therapeutic implications. Ann Med.

[CR3] Ballini M (2017). Intraneural active probe for bidirectional peripheral nerve interface.

[CR4] Beekwilder JP, Beems T (2010). Overview of the clinical applications of vagus nerve stimulation. J Clin Neurophysiol.

[CR5] BS EN 45502-1:2015. Implants for surgery. Active implantable medical devices. General requirements for safety, marking and for information to be provided by the manufacturer. London, UK: BSI Standards Limited; 2015.

[CR6] Champion HC, Skaf MW, Hare JM, Boston MA (2004). Role of nitric oxide in the pathophysiology of heart failure. The Role of Nitric Oxide in Heart Failure.

[CR7] Cracchiolo M (2021). Bioelectronic medicine for the autonomic nervous system: clinical applications and perspectives. J Neural Eng.

[CR8] Donaldson N, Baviskar P, Cunningham J, Wilson D (2011). The permeability of silicone rubber to metal compounds: relevance to implanted devices. J Biomed Mat Res A.

[CR9] Donaldson PE (1996). The essential role played by adhesion in the technology of neurological prostheses. Int J Adhesion Adhesives.

[CR10] ElAnsary M (2021). B Bidirectional peripheral nerve interface with 64 second-order opamp-Less ΔΣ ADCs and fully integrated wireless power/data transmission. IEEE J Solid-State Circuits.

[CR11] Ferraro D (2021). Implantable fiber bragg grating sensor for continuous heart activity monitoring: ex-vivo and in-vivo validation. IEEE Sens J.

[CR12] Giagka V, Demosthenous A, Donaldson N (2015). Flexible active electrode arrays with ASICs that fit inside the rat’s spinal canal. Biomed Microdevices.

[CR13] Giagka V, Eder C, Donaldson N, Demosthenous A (2015). An implantable versatile electrode-driving ASIC for chronic epidural stimulation in rats. IEEE Trans Biomed Circuits Syst.

[CR14] Habibagahi I, Jang J, Babakhani A (2022). Miniaturized wirelessly powered and controlled implants for vagus nerve stimulation.

[CR15] Jiang D, Demosthenous A (2018). A multichannel high-frequency power-isolated neural stimulator with crosstalk reduction. IEEE Trans Biomed Circuits Syst.

[CR16] Jia Y (2020). A trimodal wireless implantable neural interface system-on-chip. IEEE Trans Biomed Circuits Syst.

[CR17] Johnson RL, Wilson CG (2018). A review of vagus nerve stimulation as a therapeutic intervention. J Inflamm Res.

[CR18] Klein HU, Ferrari GMD (2010). Vagus nerve stimulation: a new approach to reduce heart failure. Cardiol J.

[CR19] Lancashire HT, Jiang D, Demosthenous A (2019). An ASIC for recording and stimulation in stacked microchannel neural interfaces. IEEE Trans Biomed Circuits Syst.

[CR20] Lionetti V (2013). Impact of acute changes of left ventricular contractility on the transvalvular impedance: validation study by pressure-volume loop analysis in healthy pigs. Plos One.

[CR21] Liu F (2022). A fully implantable opto-electro closed-loop neural interface for motor neuron disease studies. IEEE Trans Biomed Circuits Syst.

[CR22] Liu X (2012). Active books: The design of an implantable stimulator that minimizes cable count using integrated circuits very close to electrodes. IEEE Trans Biomed Circuits Syst.

[CR23] Li X (2022). System design of a closed-loop vagus nerve stimulator comprising a wearable EEG recorder and an implantable pulse generator. IEEE Circuits Syst Mag.

[CR24] Meyers EC (2018). Vagus nerve stimulation enhances stable plasticity and generalization of stroke recovery. Stroke.

[CR25] Piech DK (2020). A wireless millimetre-scale implantable neural stimulator with ultrasonically powered bidirectional communication. Nat Biomed Eng.

[CR26] Sabbah HN (2011). Vagus nerve stimulation in experimental heart failure. Heart Fail Rev..

[CR27] Shah JV (2022). A highly miniaturized, chronically implanted ASIC for electrical nerve stimulation. IEEE Trans Biomed Circuits Syst.

[CR28] Stieglitz T, Beutel H, Meyer J-U (2000). “Microflex”—A new assembling technique for interconnects. J Intell Mater Syst Struct.

[CR29] Strauss I (2023). Neural stimulation hardware for the selective intrafascicular modulation of the vagus nerve. IEEE Trans Neural Syst Rehabil Eng.

[CR30] Vallone F (2021). Simultaneous decoding of cardiovascular and respiratory functional changes from pig intraneural vagus nerve signals. J Neural Eng.

[CR31] Vanhoestenberghe A, Donaldson N (2013). Corrosion of silicon integrated circuits and lifetime predictions in implantable electronic devices. J Neural Eng.

[CR32] Wang H (2004). Cholinergic agonists inhibit HMGB1 release and improve survival in experimental sepsis. Nat Med.

[CR33] Wu Y, Jiang D, Demosthenous A (2021). A multi-channel stimulator with high-resolution time-to-current conversion for vagal-cardiac neuromodulation. IEEE Trans Biomed Circuits Syst.

[CR34] Yuan H, Silberstein SD (2015). Vagus nerve and vagus nerve stimulation, a comprehensive review: part II. Headache.

